# Acquired resistance to PI3K/mTOR inhibition is associated with mitochondrial DNA mutation and glycolysis

**DOI:** 10.18632/oncotarget.22655

**Published:** 2017-11-24

**Authors:** King Xin Koh, Gim Hwa Tan, Sarah Hong Hui Low, Mohd Feroz Mohd Omar, Min Ji Han, Barry Iacopetta, Ross Soo, Mounia Beloueche-Babari, Bhaskar Bhattacharya, Richie Soong

**Affiliations:** ^1^ Cancer Science Institute of Singapore, National University of Singapore, Singapore, Singapore; ^2^ Department of Haematology Oncology, National University Cancer Institute of Singapore, Singapore, Singapore; ^3^ School of Biomedical Sciences, The University of Western Australia, Perth, Australia; ^4^ Division of Radiotherapy and Imaging, The Institute of Cancer Research and Royal Marsden NHS Foundation Trust, Sutton, United Kingdom; ^5^ Department of Pathology, National University of Singapore, Singapore, Singapore

**Keywords:** acquired drug resistance, mitochondrial DNA mutation, glycolysis, cancer metabolism, PI3K inhibitors

## Abstract

Acquired resistance (AQR) to drug treatment occurs frequently in cancer patients and remains an impediment to successful therapy. The aim of this study was to gain insight into how AQR arises following the application of PI3K/mTOR inhibitors. H1975 lung cancer cells with EGFR T790M mutations that confer resistance to EGFR inhibitors underwent prolonged treatment with the PI3K/mTOR inhibitor, BEZ235. Monoclonal cells with stable and increased resistance to BEZ235 were obtained after 8 months treatment. These AQR clones showed class-specific resistance to PI3K/mTOR inhibitors, reduced G1 cell cycle arrest and impedance of migration following PI3K/mTOR inhibition, reduced PTEN expression and increased Akt and S6RP phosphorylation. Transcriptome analysis revealed the AQR clones had increased expression of the metabolite transporters SLC16A9 and SLC16A7, suggestive of altered cell metabolism. Subsequent experiments revealed that AQR clones possess features consistent with elevated glycolysis, including increased levels of glucose, lactate, glutamine, glucose dependence, GLUT1 expression, and rates of post-glucose extracellular acidification, and decreased levels of reactive oxygen species and rates of oxygen consumption. Combination treatment of BEZ235 with the glycolysis inhibitor 3-bromopyruvate was synergistic in AQR clones, but only additive in parental cells. DNA sequencing revealed the presence of a mitochondrial DNA (mtDNA) MT-C01 variant in AQR but not parental cells. Depletion of mitochondrial DNA in parental cells induced resistance to BEZ235 and other PI3K/mTOR inhibitors, and was accompanied by increased glycolysis. The results of this study provide the first evidence that a metabolic switch associated with mtDNA mutation can be an underlying mechanism for AQR.

## INTRODUCTION

The manifestation of acquired resistance (AQR) to drug treatment following initial response remains a major obstacle in cancer treatment [1, 2]. Recent evidence has shown that acquisition of DNA mutations in tumors can be a common mechanism for AQR, especially to molecular-targeted therapies [3]. A prominent example is the emergence of *EGFR* T790M “gatekeeper” mutations in tumors at the time of disease recurrence and following initial response to EGFR tyrosine kinase inhibitors in non-small cell lung cancer (NSCLC) patients. Other examples include the emergence of *KIT D186V/D186Y* mutations in patients treated with imatinib, and *ALK L1196M* mutations in patients treated with crizotinib.

One approach to counter AQR has been the use of signal transduction inhibitors that act downstream of the initial target of inhibition [4]. A model for this strategy is the inhibition of phosphoinositide 3-kinase (PI3K) and mammalian target of rapamycin (mTOR) signaling in EGFR inhibitor-refractory disease [5–7]. The PI3K/mTOR pathway is a major mediator of EGFR signaling [8], and the activation of this pathway has been observed in EGFR inhibitor-refractory disease [9]. Application of the PI3K/mTOR inhibitor, BEZ235, has been shown to inhibit both the *in-vitro* and *in-vivo* proliferation of cells refractory to EGFR inhibitors [7, 10]. A clinical trial has also been initiated involving the use of BEZ235 in NSCLC patients who experience a recurrence following initial response to treatment with EGFR inhibitors (NCT00620594, www.clinicaltrials.gov).

The present study was undertaken to gain insight into the mechanism by which AQR could arise following the application of PI3K/mTOR inhibitors to EGFR inhibitor-refractory lung cancer.

## RESULTS

### Generation of H1975 clones with acquired resistance to BEZ235

**Figure 1:**
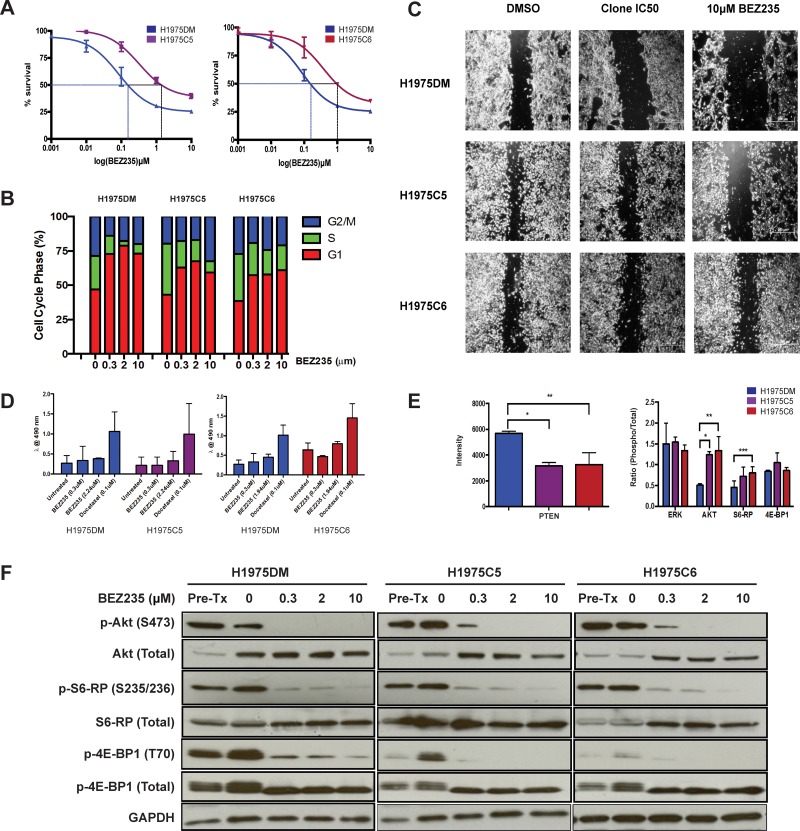
Phenotypes of H1975DM, H1975C5 and H1975C6 cells (**A**) Concentration response curves to BEZ235 at 72 h post-treatment. (**B**) Cell cycle phase distribution following 24h exposure to IC_50_ concentrations and 10μM BEZ235. (**C**) Phase contrast images of cells at 20x magnification in migration (wound-healing) assays following 24 h exposure to DMSO and IC_50_ concentrations or 10 μM BEZ235. (**D**) Apoptosis levels after 24 h exposure to IC_50_ concentrations and 10 μM BEZ235, and 0.1μM docetaxel as a positive control. (**E**) Baseline protein levels of PTEN, and ratios of phosphorylated to total ERK, AKT, S6RP and 4EBP1, determined by western blotting and densitometry. All levels were normalized to GAPDH. (**F**) Western blots of respective proteins in cells after 24h exposure to DMSO, and IC_50_ concentrations and 10μM BEZ235. ^*^ *p* < 0.05, paired *t*-test. All experiments were performed in triplicate.

The IC_50_ concentration of BEZ235 in H1975 human NSCLC cells harboring *EGFR* T790M mutations was 0.32 ± 0.13μM, consistent with previous reports [7]. H1975 cells were continuously exposed to IC50 concentrations of BEZ235 (H1975C) or DMSO (H1975DM) *in-vitro* until stable increased resistance was observed at 8 months. From the H1975C cells, 8 clones were generated through single cell colony selection and tested for sensitivity to BEZ235. Two clones with the greatest resistance to BEZ235, H1975C5 (7.2-fold) and H1975C6 (6.1-fold) (Figure 1A), were selected for further experiments. These clones retained similar levels of resistance to BEZ235 when passaged in drug-free media over 12 months (results not shown), demonstrating the AQR to BEZ235 was not transient.

### Cross-resistance to other drugs

All three cell lines retained resistance to gefitinib (IC50 > 10 μM) (Table 1), consistent with previous reports for H1975 cells [11]. H1975C5 and H1975C6 exhibited increased resistance to class IA PI3K inhibitors (GDC0941, BYL719) and a mTOR inhibitor (Ku-0063794) compared to H1975DM cells, indicating the AQR was class-specific rather than just specific to BEZ235. The three cell lines were not differentially sensitive to the MEK inhibitors PD98059 and AZD6244, inferring that the ERK pathway was not activated in AQR to BEZ235, as was hypothesized previously by others [12]. There was also no difference in sensitivity to the cytotoxic agent docetaxel, suggesting that AQR to BEZ235 does not develop through a multidrug resistance mechanism.

**Table 1:** IC_50_ concentrations of respective drugs in respective derivatives of H1975 cells

Drug	Class	H1975DM	H1975C5	H1975C6
BEZ235	PI3K/mTOR inhibitor	0.32 ± 0.13	2.29 ± 0.44^*^	1.94 ± 0.96^*^
GDC0941	PI3K inhibitor	25 ± 12	> 100	> 100
BYL719	PI3K inhibitor	6.0 ± 1.7	40 ± 25^*^	14 ± 2.5^*^
Ku0063794	mTOR inhibitor	0.64 ± 0.78	8.7 ± 0.51^*^	7.0 ± 1.5^*^
Gefitinib	EGFR inhibitor	> 10	> 10	> 10
PD98059	MEK inhibitor	> 10	> 10	> 10
AZD6244	MEK inhibitor	15 ± 1.8	19 ± 2.8	17 ± 3.1
Docetaxel	cytotoxic	< 0.001	< 0.001	< 0.001

### Cell cycle arrest, apoptosis and cell migration

Consistent with previous reports [13, 14], BEZ235 treatment of AQR and H1975DM cells led to G1 cell cycle arrest (Figure 1B) and reduced cell migration (Figure 1C), but had no significant effect on apoptosis levels (Figure 1D). When compared to H1975DM cells, the AQR clones had a reduced G1 block, reduced impedance of cell migration, but no difference in induced apoptosis levels following BEZ235 treatment.

### PI3K and mTOR pathway signaling

Compared to H1975DM cells, the AQR clones at baseline had a significantly reduced PTEN expression, increased Akt (S473) and S6RP (S235/236) phosphorylation, but no difference in 4EBP1 (T70) phosphorylation (Figure 1E). Exposure to BEZ235 reduced the phosphorylation of Akt, S6RP and 4EBP1 in H1975DM and the AQR cells (Figure 1F), indicating that altered sensitivity to target inhibition was unlikely to be a mechanism of AQR to BEZ235.

### DNA mutation

Targeted next generation sequencing was used to identify likely somatic mutations in regions of high mutation frequency in 50 cancer-related genes. *EGFR* (L858R, T790M), *TP53* (R273H) and *CDKN2A* (E69X) mutations were detected in all three H1975-derived cell lines, consistent with previous reports [15]. A previously reported G118D mutation in exon 2 of *PIK3CA* could not be confirmed, as the assay did not interrogate this exon. A previously unreported I391M mutation in exon 6 of *PIK3CA* was also detected. No other mutations were detected.

### Gene expression

**Figure 2:**
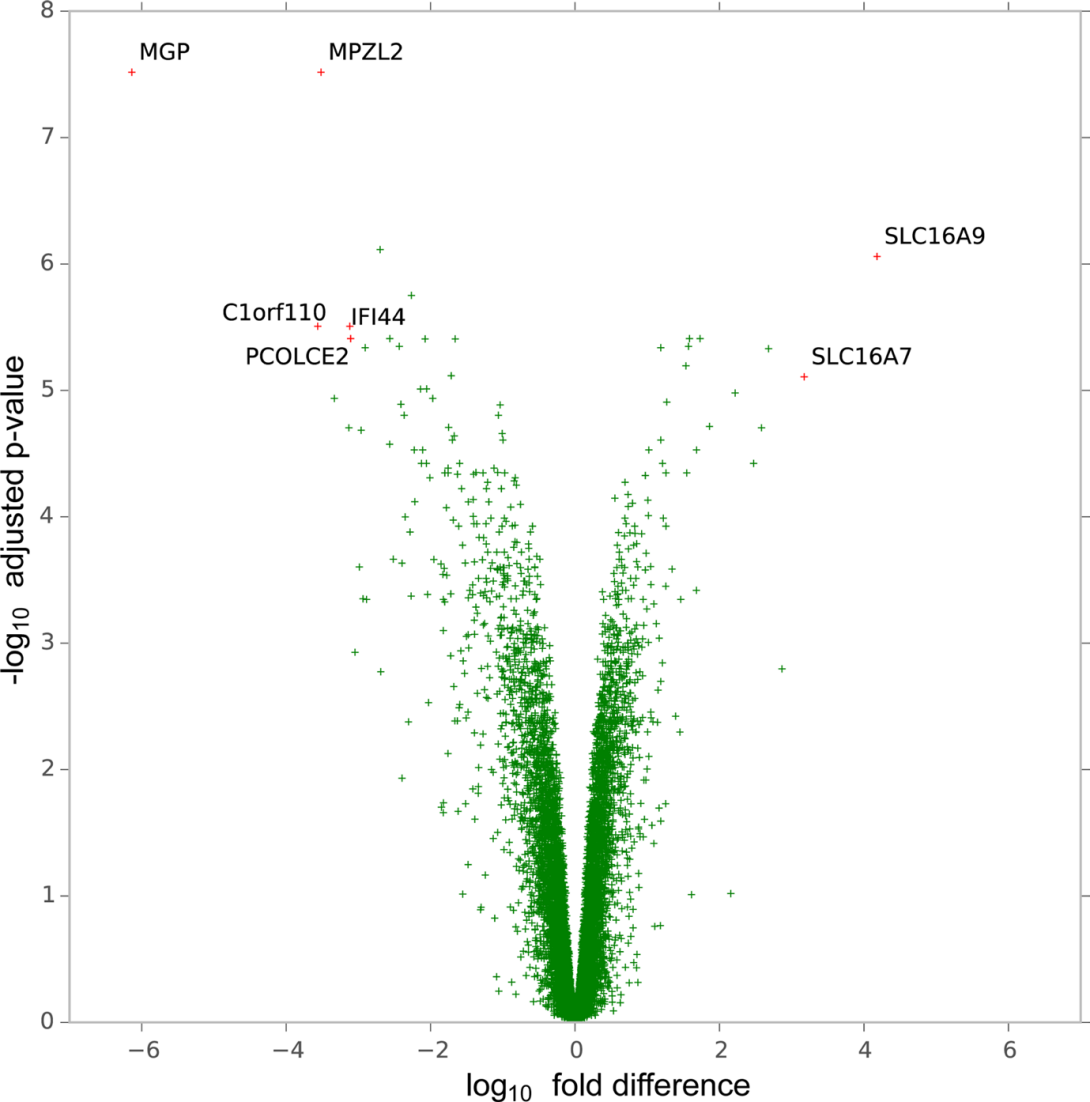
Volcano plot of genes according to the magnitude (fold-difference, x-axis) and significance level (-log_10_ adjusted *p*-value, y-axis) of their difference in expression in H1975C5 and H1975C5 cells compared to H1975DM cells from gene expression array analysis

Compared to H1975DM cells, 997 genes were differentially expressed with an adjusted *p* < 0.05 and at least 2-fold difference in AQR clones (Figure 2A, Supplementary Table 1). Of these, the monocarboxylate transporters (MCT), *SLC16A7* (*MCT2*) (adjusted *p* = 7.82 × 10^-6^, 3.2-fold increase) and *SLC16A9* (*MCT9*) (adjusted *p* = 8.73 × 10^-7^, 4.2-fold increase) showed the greatest increase in expression in AQR clones. *MCT2* and *MCT9* mediate the transport of various metabolic byproducts, including lactate [16] and carnitine [17], respectively. Since lactate is the end product of glycolysis [18], and carnitine is involved in mitochondrial lipid transport [19] and acetyl-CoA equilibrium [20], the observed increase in expression of *MCT2* and *MCT9* prompted us to investigate the metabolic activity of the cells.

### Glucose metabolism and cellular energetics

**Figure 3:**
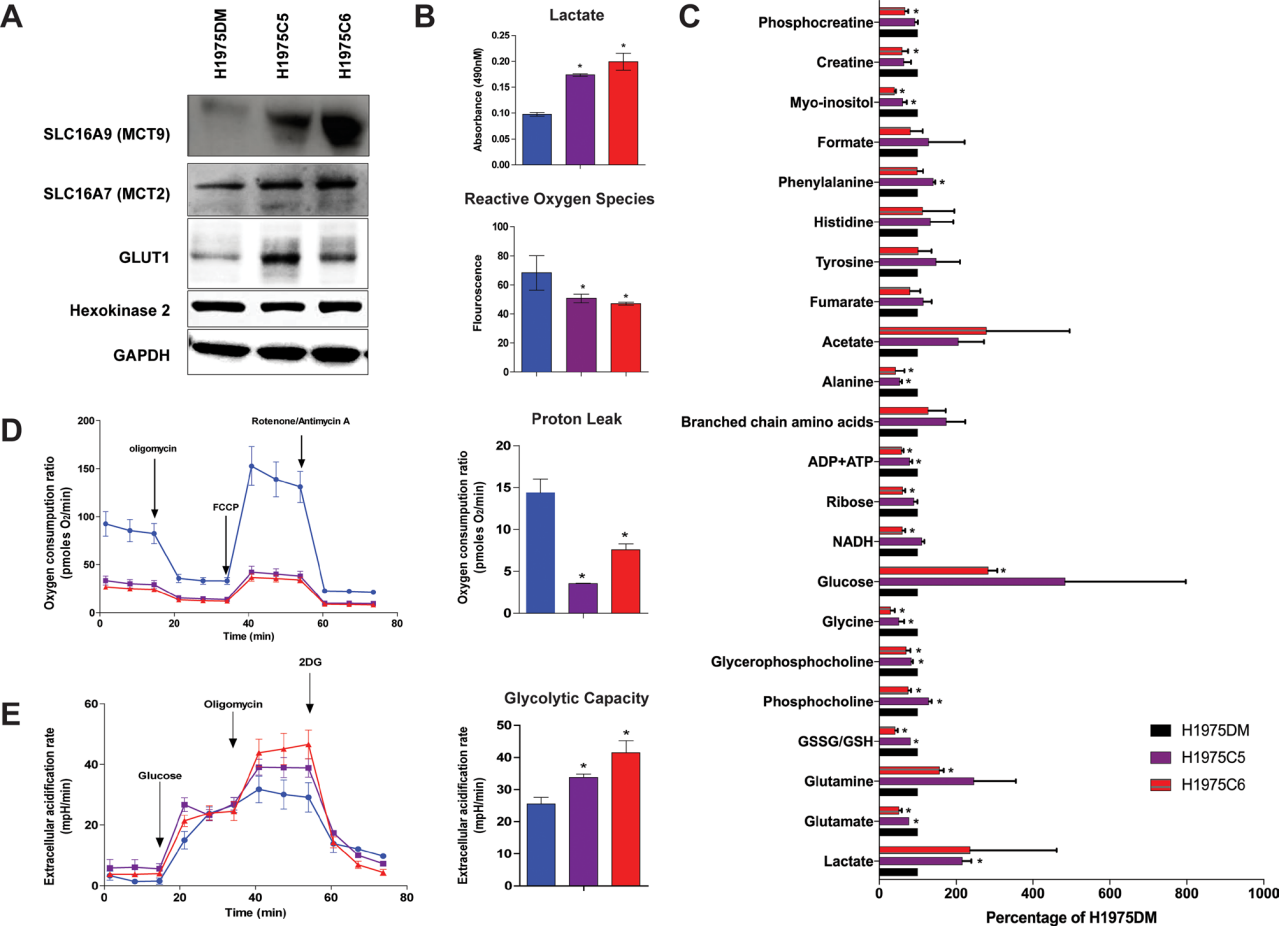
Protein levels and metabolism of H1975DM, H1975C5 and H1975C6 cells (**A**) Representative immunoblot of MCT9, MCT2, GLUT1 and Hexokinase 2, and GAPDH as a loading control. (**B**) Baseline levels of lactate and reactive oxygen species. (**C**) Baseline metabolite levels according to NMR analysis, relative to levels in H1975DM cells. (**D**) Kinetic OCR response to oligomycin (for determining ATP coupled respiration), carbonyl cyanide-p-trifluoromethoxy-phenylhydrazone (FCCP) (to establish maximal respiratory capacity) and rotenone (to examine mitochondrial respiration). Proton leak calculation derived from the difference in measurement 12 and measurement 4. (**E**) Kinetic extracellular acidification rate response to glucose, oligomycin and 2-DG. Glycolytic capacity is the difference between extracellular acidification rate of measurement 9 and that of measurement 3. Displayed are the mean ± SD from triplicate analysis. ^*^ indicates *p* < 0.05, paired *t*-test.

Western blot analysis confirmed the increased expression of MCT2 and MCT9 in AQR clones compared to H1975DM cells (Figure 3A). Consistent with increased glycolysis, GLUT1 levels were also higher in AQR clones, although no differences were observed for hexokinase 2 levels. In glucose dependency experiments using crystal violet staining, H1975DM cells were able to grow in the absence of glucose, whereas AQR clones did not (results not shown). Compared to H1975DM cells, the AQR clones also showed increased extracellular lactate levels and reduced reactive oxygen species (Figure 3B), consistent with increased glycolysis.

Analysis of the intracellular levels of 22 metabolites by ^1^H NMR spectroscopy presented further evidence of increased glycolysis, including significantly increased lactate, glutamine, and glucose levels, and reduced glutamate, glutathione, NADH, and ADP+ATP levels in at least one or both AQR clones compared to H1975DM cells (Figure 3C). Consistent with PI3K/mTOR inhibition [21], reductions in phosphocholine and glycerophosphocholine were observed, although H1975C5 cells also displayed increased phosphocholine. Increased phenylalanine and reduced glycine, ribose, alanine, myo-inositol, creatine and phosphocreatine levels were also observed.

Seahorse XFp extracellular flux analysis showed the AQR clones had lower basal oxygen consumption ratios compared to H1975DM cells (Figure 3D). Assessment of the ratios after mitochondrial uncoupling by carbonyl cyanide-p-trifluoromethoxy-phenylhydrazone revealed the mitochondrial respiratory capacity and proton leak was also lower in AQR clones. The extracellular acidification ratio after glucose addition, representing the glycolytic capacity, was higher in AQR clones compared to H1975DM cells (Figure 3E).

### Mitochondrial DNA sequencing

**Figure 4:**
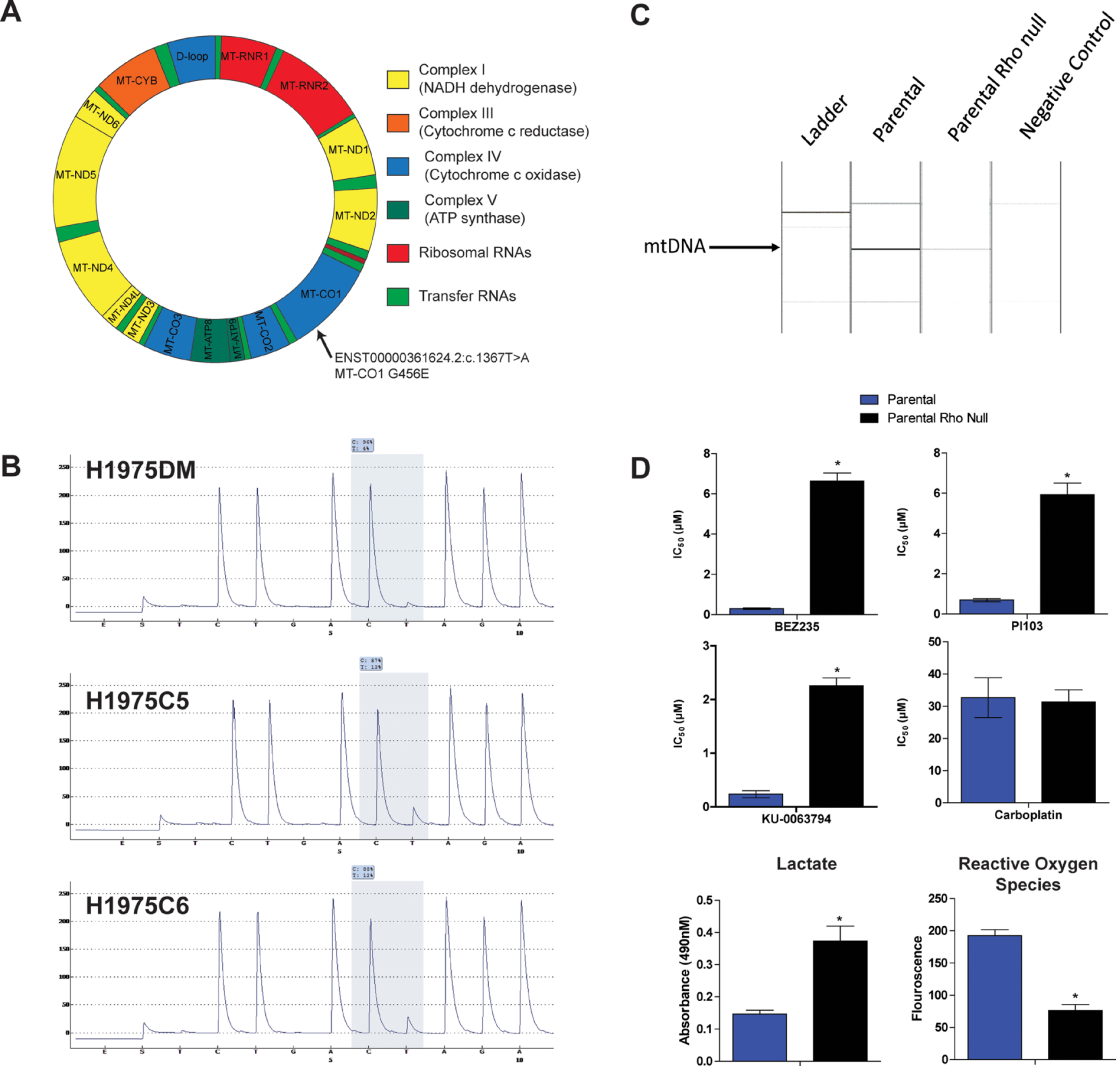
(**A**) Representation of somatic mitochondrial DNA variants detected only in AQR clones in MT-CO1 gene. (**B**) Validation of the ENST00000361624.2: c.1367T>A variant by pyrosequencing. (**C**) PCR verification of ρ(o) status of H1975ρ(o) cells compared with parental H1975 cells. (**D**) Bar charts showing IC50 values of BEZ235, PI103, KU-0063794 and carboplatin in H1975 (blue columns) and H1975ρ0 (black columns) cells. Also shown are the levels of extracellular lactate and reactive oxygen species in H1975 and H1975ρ0 cells. ^*^p < 0.05, paired *t*-test.

The high glycolysis and low mitochondrial respiration rates of the AQR clones prompted sequencing of mitochondrial DNA (mtDNA). Based on the Cambridge mitochondrial reference sequence, 20 mtDNA variants were identified in all three cell lines (Supplementary Table 2). Two variants were found in both AQR clones but not in H1975DM cells, namely a coding variant in mitochondrial encoded cytochrome c oxidase I (*MT-CO1*) (ENST00000361624.2: c.1367T>A, G456E) and a non-coding variant in mitochondrial encoded 16S RNA (MT-RNR2) (ENST00000387347: c.819A>C) (Figure 4A). The coding *MT-CO1* G456E variant was confirmed by pyrosequencing (Figure 4B).

### Drug sensitivity of H1975 Rho null cells

H1975ρ0 cells lacking mtDNA were generated from parental H1975 cells as described in the Methods section and were verified to lack mtDNA by PCR analysis (Figure 4C). H1975 cells cultured in the presence of ethidium bromide but without additional pyruvate and uridine did not grow (results not shown), indicating the preparation process had led to a loss of mitochondrial function. H1975ρ0 cells were less sensitive to PI103, BEZ235 and KU-0063794 compared to parental cells, supporting the notion that mitochondria are a determinant of sensitivity to these drugs (Figure 4D). However, no difference was observed in the sensitivity of H1975ρ0 and parental cells to carboplatin, indicating the influence of mitochondrial function was class-specific. H1975ρ0 cells also had increased lactate and decreased reactive oxygen species levels compared to parental cells, consistent with a shift in energy generation from oxidative phosphorylation to glycolysis in H1975ρ0 cells.

### Combination of 3-Bromopryruvate and BEZ235 Treatment

To further investigate the AQR phenotype, sensitivity to the glycolysis inhibitor 3-Bromopyruvate (3BP) was assessed. H1975C5 (IC_50_= 31 ± 2.8 μM) and H1975C6 (29 ± 3.2 μM) AQR clones were significantly more sensitive to 3BP compared to H1975DM cells (75 ± 6.2 μM) (Supplementary Figure 1A). To examine whether 3BP could re-sensitize AQR clones to BEZ235, a fixed-ratio combination treatment of 3BP and BEZ235 was performed. The combination of 3BP and BEZ235 was synergistic in their effect on cell proliferation in H1975C5 (CI = 0.56 ± 0.01) and H1975C6 (CI = 0.47 ± 0.01) cells, but additive in H1975DM (CI = 1.21 ± 0.02) cells (Supplementary Figure 1B). Further investigation indicated there was no difference in apoptosis levels between the cell lines following combination treatment (Supplementary Figure 1C). Instead, significantly higher LC3B levels, suggestive of increased autophagosomes [22], were observed in the AQR clones compared to H1975DM cells treated with the combination and 3BP alone. Intracellular ATP levels in AQR clones treated with the combination were also decreased compared to baseline levels, although this was also observed in H1975DM cells.

## DISCUSSION

This study was aimed at bringing further insight into general mechanisms of AQR in the context of the application of PI3K/mTOR inhibitors to EGFR inhibitor-refractory lung cancer. However, the progress of PI3K/mTOR inhibitors into the clinic has recently slowed due to issues with safety and efficacy [5, 23]. Indeed, the clinical trial involving BEZ235 in EGFR inhibitor-refractory disease closed prematurely due to a high frequency of unanticipated adverse events [24, 25]. Meanwhile, third-generation EGFR inhibitors have since been developed and shown to provide a survival benefit in the refractory setting [26, 27]. The relevance of the clinical indication for which this study was conducted has therefore been somewhat diminished.

Nonetheless, this study has uncovered a new and potentially important mechanism of AQR involving a switch to a glycoytic phenotype associated with mtDNA mutation. It has also provided further information on potential phenotypes of AQR to PI3K/mTOR inhibitors. Significant interest in these agents remains due to reports of clinical activity in certain individuals and indications [28], and to the high frequency of PI3K/mTOR aberrations in cancer [23]. A broader understanding of the activity and isoform specificity of these inhibitors may allow more focused application, and hence a greater realization of their potential [29].

Genome-wide gene expression analysis provided the initial indication for a metabolic switch in AQR cell lines (Figure 2). Sequencing of 50 cancer-related genes determined that no new mutations were acquired in genes such as *EGFR*, *PIK3CA*, *KRAS* and *TP53*. Evidence for a switch to increased glycolysis and reduced oxidative phosphorylation in the AQR phenotype was subsequently obtained using several experimental approaches. These included an analysis of 22 metabolites by NMR spectroscopy, GLUT1 expression, lactate and reactive oxygen species levels, glucose dependency, rates of oxygen consumption and extracellular acidification (Figure 3), and combination treatment with 3-BP (Supplementary Figure 1).

The finding of mtDNA mutations unique to AQR cells (Figure 4) provides further insight into the development of AQR. Two mtDNA mutations were observed, one of which occurred in the coding region of *MT-CO1* (G456E) and was verified by pyrosequencing. *MT-CO1* encodes a protein found within complex IV of mitochondrial redox carriers which catalyzes the reduction of oxygen to water [30]. This complex is a major regulator of oxidative phosphorylation [31], and *MT-CO1* mutations have previously been associated with weak oxidative phosphorylation in the settings of oxidative stress [32].

A sequence of events resulting from prolonged exposure to BEZ235 and leading to AQR can be hypothesized from the above findings. mTOR can be localized to the mitochondrial membrane, and its inhibition has been shown to influence mitochondrial membrane potential, oxygen consumption and ATP synthesis [33, 34]. Hence, prolonged inhibition of mTOR by BEZ235 could have interfered with mitochondrial potential, leading to mtDNA mutation and mitochondrial dysfunction. In the environment of mitochondrial dysfunction, clones that increased their glycolysis rate to compensate for the impaired oxidative phosphorylation were then able to out-compete other cells for energy production and resources. These changes included the ability to efflux elevated lactate resulting from glycolysis and excess carnitine no longer required for AcetylCoA, as can be inferred from the overexpression of *SLC16A4* and *SLC17A9* (Figure 2), respectively. These changes also included increased glutamine levels for anaplerosis, as observed by NMR spectroscopy (Figure 3C). The changes in energy requirements and pathways for cell viability, and lower dependence on mitochondrial function may have led to a lower dependence on mTOR in its roles of sensing cellular nutrient, oxygen and energy levels and integrating growth factor signaling with glycolysis and oxidative phosphorylation [35]. Through such events, the AQR clones would have had a reduced reliance on mTOR signaling for their survival, thereby manifesting in their observed AQR to PI3K/mTOR inhibitors. In support of this hypothesis, elimination of mitochondrial function in H1975 cells (H1975ρ0 cells) led to increased glycolysis, reduced oxidative phosphorylation, and increased class-specific resistance to PI3K/mTOR inhibitors (Figure 4).

To our knowledge, only one other study has investigated AQR to PI3K/mTOR inhibitors. Using genome-wide methylation analysis, Qian et al. (2015) [36] observed that AQR to BEZ235 in nasopharyngeal cancer cells was associated with increased DNA hypermethylation and suppression of *PTEN* and *PPP2R2B* expression, resulting in activation of the AKT/mTOR and PDK1/MYC pathways. Treatment with inhibitors of DNA methyltransferases increased the sensitivity of the AQR cells to BEZ235, leading the authors to conclude that DNA hypermethylation was a key modulator of AQR. Consistent with this study, reduced PTEN, along with elevated pAKT, pmTOR and pS6 expression, was observed in the current study (Figure 1). Whereas Qian et al. reported an increased G2M cell cycle distribution, a reduced G1 arrest post-BEZ235 treatment was observed in the current study (Figure 1B). A phenotype of increased migration was observed in the current study (Figure 1C), consistent with the increased invasion reported by Qian et al., and possibly related to the invasive properties associated with elevated lactate [18] (Figure 3). In both studies, reductions in pAKT, pS6 and p4EBP expression (Figure 1F) were also observed in AQR cells following BEZ235 treatment, indicating the resistant phenotype did not result from the loss of BEZ235 activity, but rather through alternative mechanisms. Taken together, the present findings and those of Qian et al. appear to be complementary. The current results shed light on how the changes observed in AQR cells may have been initiated, propagated and ultimately manifested, while those of Qian et al. demonstrate how the competitive characteristics of these cells may have developed through hypermethylation.

In conclusion, the results of this study highlight a mechanism by which AQR could develop following a switch in metabolism associated with mtDNA mutation. This expands our understanding of the ways in which AQR can develop, that have included altered drug intake and efflux, altered drug metabolism, alteration of drug targets through mechanisms such as target gene mutation and amplification, activation of redundant pathways, and impaired apoptosis [1]. Such insights provide a rationale for novel approaches to combat AQR, including the use of drugs that manipulate metabolism [37]. Indeed, AQR cells were found here to be sensitive to the glycolysis inhibitor 3-BP, and combination treatment with BEZ235 was synergistic in these cells (Supplementary Figure 1). However, additional work is required to evaluate whether the effects observed here are also valid in other *in-vitro*, *in-vivo* and clinical settings, and whether the AQR and corresponding interventional principles apply to other drugs or metabolic inhibitors. The observation of mtDNA mutations also warrants further investigation into their involvement in AQR, and possibly also with intrinsic resistance and tumorigenesis [38].

## MATERIALS AND METHODS

### Cells

H1975 cells were obtained from American Type Culture Collection (Manassas, VA) and maintained in RPMI 1640 (Thermo Fisher Scientific, Waltham, MA) supplemented with 10% fetal bovine serum (Thermo Fisher Scientific) and 1% antimycotic/antibiotic solution (Gemini Bio-Products, West Sacramento, CA) at 37°C in a humidified atmosphere containing 5% CO_2_. For the initiation of experiments, cells in the exponential growth phase were used.

### Compounds

BEZ235 was kindly provided by Novartis Pharma AG (Basel, Switzerland). Docetaxel was obtained from Sigma Aldrich (St. Louis, MO), gefitinib, and Ku0063794 from Biovision (Milpitas, CA), and PD-98059, GDC-0941, and BYL719 from Cayman Chemicals (Denver, CO). All compounds were diluted to stock solutions and stored according to supplier recommendations.

### Drug sensitivity analysis

Approximately 3,000 cells in 90 μl of medium were seeded in individual wells of 96 well plates (Nunc, Rochester, NY). Compounds were diluted to graded concentrations in culture media and 10 μl of respective aliquots were added to respective wells 24 hours after seeding. A total of 20 μl of CellTiter 96 Aqueous One Solution Cell Proliferation reagent (Promega, Madison, WI) was added to each well and incubated for 1.5 hours. Absorbance was measured at 490 nm using Tecan Infinite 200 Pro microplate reader (Tecan, Mannedorf, Switzerland). An IC_50_ value for each compound, representing the concentration of compound that inhibited cell proliferation to 50% of vehicle controls, was calculated as previously described [39].

Drug combination analysis was evaluated using the median-effect equation and combination index (CI) method of Chou and Talalay [40]. Briefly, two compounds were added at fixed ratios of their respective IC_50_ concentrations for 72 hours. The CI was then determined using the formula CI = [(D)1/(Df)1 + (D)2/(Df)2] + [(D1)(Df)2/(Df)1(Df)2], where (D)1 and (D)2 are the concentrations of the combination required to produce the fraction unaffected, and (Df)1 and (Df)2 are the concentrations of the individual drugs required to produce the fraction unaffected. Median-effect plots with linear regression coefficients (R^2^) < 0.95 were excluded. CI values with the non-exclusive assumption were used for reporting. CI values of less than 1, between 1-2 or greater than 2 were considered to indicate synergism, additivity or antagonism, respectively.

### Cell cycle distribution analysis

Cells were washed with ice cold phosphate-buffered saline (PBS), trypsinized and fixed with 70% ethanol at 4°C. The cells were incubated in a staining solution (1 × PBS, 100U/mL RNase A, 40U/mL propidium iodide) for 24 hours. Cell counting was then performed using the BD LSRII flow cytometer (BD Biosciences, Franklin Lakes, NJ).

### Cell apoptosis analysis

Approximately 3,000 cells were seeded per well in 96-well plates and allowed to adhere for 24 hours. Cells were exposed to appropriate compounds for 24 hours and detection of nucleosomes was then performed using the Roche Cell Death ELISA Detection Kit (Roche, Mannheim, Germany) according to manufacturer recommendations.

### Cell migration analysis

Approximately 2 × 10^5^ cells were plated per well in 6-well tissue culture plates and allowed to proliferate to 90% confluence. A scratch in each well was made using a 200μl pipette tip. Concentrations of BEZ235 (equimolar, equitoxic and 5×IC50) and DMSO as a control were added in respective wells. Images of the cells were taken 24 hours after drug exposure using a Axio Vert.A1 microscope (Zeiss, Oberkochen, Germany).

### Protein expression analysis

Cells were washed with ice cold PBS, and lysed with cell lysis buffer (Cell Signaling Technology, Beverly, MA). Protein concentrations were determined using the BCA protein estimation assay (Thermo Fisher Scientific). Equal amounts of protein were combined with 5× sample buffer (250mM Tris-HCl pH6.8, 10% SDS, 30% glycerol, 5% β-mecaptoethanol, and 0.02% bromophenol blue) at a 1:4 ratio and separated on a 4–20% Tris-glycine pre-cast gel (Thermo Fisher Scientific). Proteins were transferred to a 0.45μm nitrocellulose membrane before blocking with 5% milk in TBS-T (20mM Tris, 150mM NaCL, 0.01% Tween-20) for 60 mins, and then incubation with specific antibodies. The blots were incubated with a HRP-linked secondary antibody, resolved with the RPN2232 chemiluminescence reagent (GE Healthcare, Little Chalfont, UK) and visualized using X-ray film on the ImageQuant LAS500 (GE Healthcare).

### Targeted genomic DNA sequencing

DNA was extracted from cells using the DNeasy Blood and Tissue kit (Qiagen) and analyzed for DNA variants in 50 genes using the Ion AmpliSeq Cancer Hotspot Panel v2 assay on the Ion Torrent PGM (Thermo Fisher Scientific). The genes analyzed were *ABL1, AKT1, ALK, APC, ATM, BRAF, CDH1, CDKN2A, CSF1R, CTNNB1, EGFR, ERBB2, ERBB4, EZH2, FBXW7, FGFR1, FGFR2, FGFR3, FLT3, GNA11, GNAS, GNAQ, HNF1A, HRAS, IDH1, IDH2, JAK2, JAK3, KDR, KIT, KRAS, MET, MLH1, MPL, NOTCH1, NPM1, NRAS, PDGFRA, PIK3CA, PTEN, PTPN11, RB1, RET, SMAD4, SMARCB1, SMO, SRC, STK11, TP53, and VHL*. Sequencing was performed using the Ion Ampliseq Sequencing 2.0 kit and Ion 318 chips. Data analysis was performed using Ion Torrent Suite V3.6.2 with the IT Variant Caller Plugin, V3.6. DNA variants were filtered for those that had a quality score greater than 200, were non-synonymous, and had a minor allele frequency of less than 5% in the 1000 Genomes Database.

### Gene expression analysis

Approximately 5.0 × 10^5^ cells were seeded in T25 flasks in triplicate, incubated for 24 hours and then washed with PBS and trypsinized. RNA was extracted from the cells using the Qiagen RNeasy Blood and Tissue Kit (Qiagen). Amplified cDNA was generated from 200 ng RNA using the Applause WT-Amp ST system (NuGEN, San Carlos, CA) and analysed using Affymetrix Human Gene 1.0ST array on the Genechip Fluidics Station 450 FS450 (Thermo Fisher Scientific). CEL files were loaded into the R Bioconductor environment using the oligo package [41], normalized using the Robust Multi-Array Average method with the default settings [42], and assessed using the Linear Models for Microarray method [43].

### Metabolite analysis

Cells were washed in cold saline and mixed with equal volumes of cold methanol, chloroform and water. Lyophilized samples of the water-soluble phase were reconstituted in 540μl of a D_2_O solution containing 0.075% (w/v) 3-(trimethylsilyl) propionic-2,2,3,3-d4 acid as internal reference for analysis by ^1^H NMR. The ^1^H NMR spectra were acquired at room temperature on a 500 MHz Bruker spectrometer (Billerica, MA) using a 30° flip angle, a 1s repetition delay, a spectral width of 13 ppm and 64 K data points under conditions of water signal suppression. Spectra were processed using MestRe-C version 4.9.9.6 (University of Santiago de Compostela, Spain) and the content was determined by peak integration, normalized relative to the internal standard and corrected for cell number per sample.

### Mitochondrial respirometry

Mitochondrial oxygen consumption ratio and extracellular acidification rate analysis was performed using Seahorse XFp Extracellular Flux Analyzer (Agilent). Cells were cultured in XFp Miniplates at a concentration of 10,000 cells/well in RPMI medium. After 24 h, cells were washed twice with 180 μl of assay medium and incubated at 37°C for one hour. For oxygen consumption ratio, cells were analyzed using the XFp Mito Stress Test Kit. Measurements were taken at baseline, and following addition of 1μM oligomycin, 0.5 μM carbonyl cyanide-p-trifluoromethoxy-phenylhydrazone and 0.5 μM rotenone/antimycin A. For extracellular acidification rate, cells were analysed using the XFp Glycolysis Stress Test Kit. Measurements were taken after addition of 10 mM Glucose, 1μM oligomycin and 50 mM 2-deoxyglucose.

### Mitochondrial DNA sequencing

DNA was extracted from the cells using the DNeasy Blood and Tissue kit (Qiagen). Mitochondrial DNA was amplified from 200ng DNA using the REPLI-g Mitochondrial DNA Kit (Qiagen). Subsequently, 4nM of mitochondrial DNA were sequenced using the Illumina MiSeq Desktop Sequencer (Illumina). Sequencing reads were aligned to the Revised Cambridge Reference Sequence (rCRS) (gi|251831106|ref|NC_012920.1) [44] using BWA v0.7.12 [45]. An additional alignment for insertions and deletions was also performed using the GATK IndelRealigner v3.4-0 (10.1101/gr.107524.110). Genomic variations were identified by referencing the rCRS sequence using the GATK unified genotyper (10.1101/gr.107524.110). Variants were annotated using Variant Effect Predictor (VEP) perl script version 80 [46] that references the Ensembl release 80 database.

For confirmation of variants, 100 ng of mitochondria DNA underwent pyrosequencing using the PyroMark Q24 system (Qiagen). The PCR mix comprised 25 μl of 100 ng DNA, 2.5 μl of PCR buffer with MgCl_2_, 0.5 μl of dNTP mix, 0.2 μl of Taq Polymerase (Roche), and 2.5 μl of forward and 2.5 μl of reverse primer (IDT, Coralville, IA). Thermal cycling comprised 95°C for 4min, followed by 40 cycles of 30 seconds at 94°C, 30 seconds at 65°C and 30 seconds at 72°C, before incubation at 72°C for 7 min. The primers for *MT-CO1* were Forward: 5′-TTC TTC CCA CAA CAC TTT CTC G-3′, Reverse: 5′-GGG CAT CCA TAT AGT CAC TCC A-3′ and the pyrosequencing sequence was CTA C/TA GAT GAT AGG ATG TTT CAT GTG GTG. All samples were run in triplicate.

### Generation of H1975 cells lacking mitochondrial DNA

H1975ρ0 cells lacking mitochondrial DNA were generated as previously described [47]. Briefly, cells were cultured for 1 month in DMEM media containing 25mmol/L glucose supplemented with 10% fetal bovine serum, 50ng/ml ethidium bromide, 50 ng/ml uridine, 1mM sodium pyruvate and 1× antibiotic antimycotic solution (Sigma-Aldrich, St. Louis, MO). For verification, DNA was extracted from the cells using the DNeasy Blood and Tissue kit (Qiagen) and underwent PCR analysis for nuclear and mitochondrial DNA. The PCR mix comprised 100 ng DNA, 2.5 μl of PCR buffer with MgCl_2_, 0.5 μl of DNTP mix, 0.2 μl of Taq Polymerase (Roche), and 2.5 μl of forward and reverse primer (IDT) in a total volume of 25μl. The primers were as follows: Nuclear DNA Forward: 5′-CAT TGC TCC TCC TGA GCG CAA-3′, Nuclear DNA Reverse: 5′-GCT GTC ACC TTC ACC GTT CCA-3′, Mitochondrial DNA Forward: 5′-ACA ATA GCT AAG ACC CAA ACT GGG-3′, Mitochondrial DNA Reverse: 5′-CCA TTT CTT GCC ACC TCA TGG GC-3′. Thermal cycling comprised 95°C for 5 min, followed by 40 cycles of 1 min at 94°C, 1 min at 60°C (nuclear DNA) or 63°C (mitochondrial DNA) and 1 min at 72°C, before incubation at 72°C for 10 min. PCR product was analysed using the QIAxcel DNA Screening Kit on the QIAxcel Advanced System (Qiagen).

## SUPPLEMENTARY MATERIALS FIGURES AND TABLES







## Abbreviations

3-BP: 3-bromopyruvate
AQR: acquired resistance
mtDNA: mitochondrial DNA
