# Supplementary material for: Acquired resistance to PI3K/mTOR inhibition is associated with mitochondrial DNA mutation and glycolysis
Source: Oncotarget. 2017 Nov 24;8(66):110133–44. doi: 10.18632/oncotarget.22655 (PMC5746370; doi:10.18632/oncotarget.22655)
Supplement: Supplementary file 1 [file oncotarget-08-110133-s001.pdf]

Acquired resistance to PI3K/mTOR inhibition is associated with mitochondrial DNA mutation and glycolysis

SUPPLEMENTARY MATERIALS

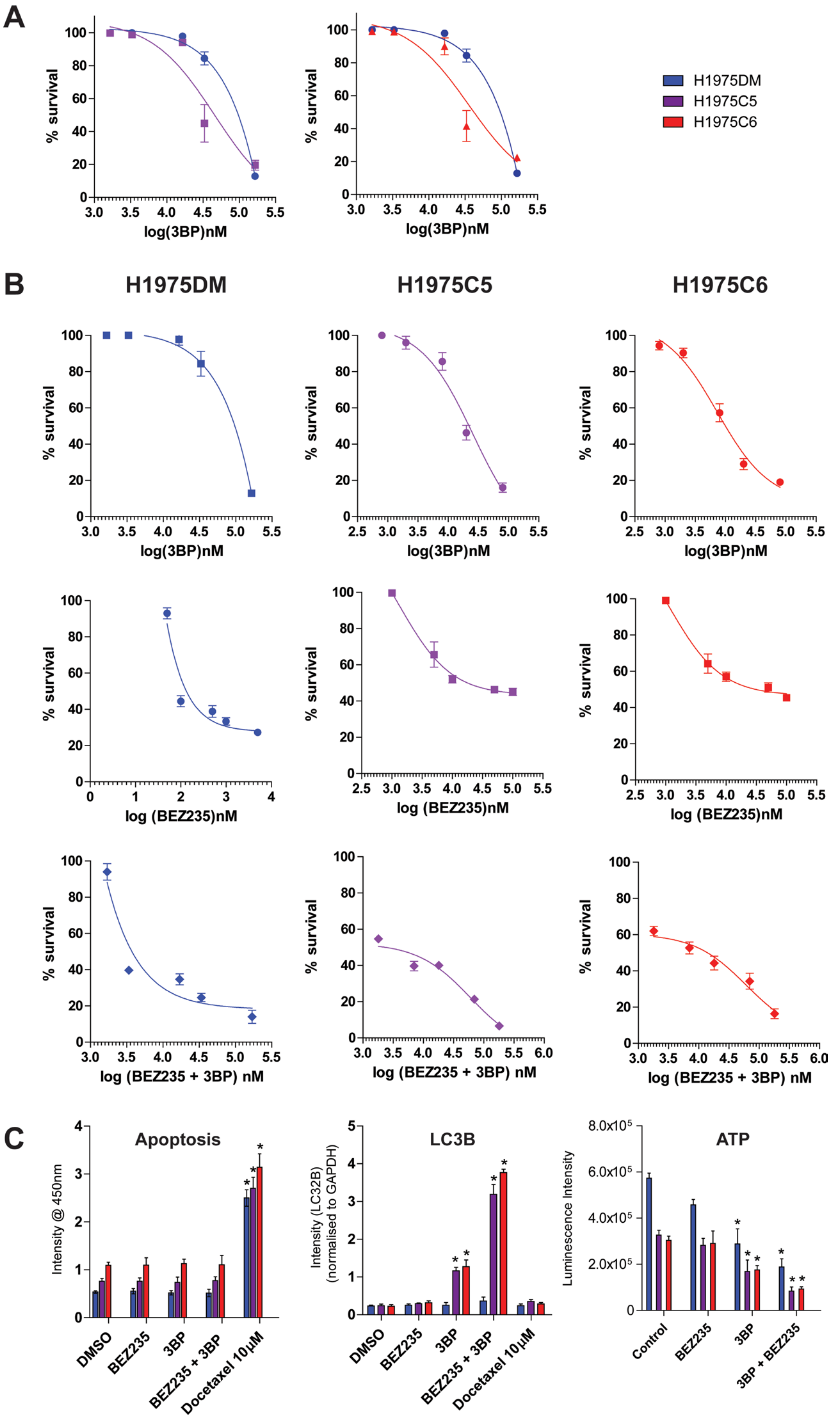

Supplementary Figure 1: Effect of treatment with BEZ235 alone, 3-bromopyruvate (3BP) alone, and 3BP and BEZ235 in combination in H1975DM, H1975C5, and H1975C6 cell lines. (**A**) Concentration response curves of 3BP. (**B**) Concentration response curves for 3BP (upper panels), BEZ235 (middle panels) and the fixed-ratio combination of BEZ235 and 3BP (lower panels). (**C**) Apoptosis, LC3B and ATP levels 24h post-exposure to IC50 concentrations of BEZ235, 3BP or their combination. $^{*}p < 0.05$ to levels in control-treated cells for each cell line, paired *t*-test.

Supplementary Table 1: Gene expression levels and differences according to genome-wide gene expression array analysis. See Supplementary_Table_1

Supplementary Table 2: Mitochondrial DNA variants detected by next generation sequencing of mitochondrial DNA, according to their reported and predicted effects, and presence in H1975DM, H1975C5, and H1975C6 cells. See Supplementary_Table_2
